# The Subjective and Objective Improvement Using Chiropractic Biophysics® Protocols

**DOI:** 10.7759/cureus.50533

**Published:** 2023-12-14

**Authors:** Jason W Haas, Thomas Woodham, Paul A Oakley, Miles O Fortner, Deed Harrison

**Affiliations:** 1 Research, Chiropractic BioPhysics (CBP) Non-Profit, Windsor, USA; 2 Chiropractic, Chiropractic BioPhysics, Gillette, USA; 3 Chiropractic, Western Plains Chiropractic, Gillette, USA; 4 Kinesiology and Health Science, York University, Toronto, CAN; 5 Chiropractic, Private Practice, Newmarket, CAN; 6 Chiropractic, Chiropractic BioPhysics (CBP) Non-Profit, Windsor, USA

**Keywords:** failed back surgery syndrome (fbss), posture correction, chronic low back pain, pedicle screw fixation, persistent spine pain syndrome

## Abstract

The aim of this study is to describe the Chiropractic BioPhysics^®^ (CBP®) (Chiropractic BioPhysics, Eagle, USA) technique in alleviating the persistent spine pain syndrome (PSPS) and dysfunction in a 50-year-old female who suffered for many years. The purpose of this study is to provide clinicians with a potential treatment option for failed back surgery syndrome (FBSS) and PSPS that doesn't respond to other treatments. The patient did not receive benefits from pharmaceutical and conservative therapies following a low back lifting injury in 2004. After several years of suffering from widespread spinal pain and dysfunction, she received a lumbosacral pedicle screw surgical fixation. The initial surgery was unsuccessful and a follow-up revision and expansion of the fusion failed to alleviate the pain and dysfunction as well. After treatment using CBP, the patient received subjective, objective, and radiographic improvements with long-term stability measured at follow-up. Given that spine pain and low back pain are the number one cause of disability in the world, having economical, repeatable, and measurable techniques to improve even difficult cases is important for astute clinicians treating spine pain.

## Introduction

Low back pain (LBP) and the consequences of chronic pain and disability are the main contributors to the global burden of disease (GBD). LBP has numerous causative factors including trauma, degeneration, chronic and repetitive stress injuries, mental health illnesses, and morphological and anatomical causes/cofounders [1,2]. Surgery is a frequent conventional treatment for LBP and disability, and pedicle screw fixation is a common technique [3]. Although the majority of pedicle screw fixation does not have complications, the complications can lead to significant pain and further disability for some patients; indeed, failed back surgery syndrome (FBSS) often requires lifetime drug treatment and has recently been reclassified as persistent spine pain syndrome (PSPS) and can frequently lead to more invasive and higher risk surgical revisions and re-operations [4,5].

PSPS is a serious concern for patients, clinicians, third-party payors, and therapists [6-9]. Following spinal surgery patient-reported outcomes (PROs) may worsen in the short term while the fusion/instrumentation is stabilizing but should improve in the long term as tissues heal [3]. Worsening of health-related quality of life (HRQoL) measures after a one-year period is a cause for concern and warrants further diagnostic examination including imaging, orthopedic and neurologic tests, and possibly more invasive diagnostics such as discogram and MRI with contrast agents [7]. Serious complications such as cancer, infection, vascular disease, and mental illness must be differentially diagnosed [10]. A patient who does not improve following the initial surgical procedure can expect a worsening of HRQoL, often increased pain and disability over time [6,7]. Many patients can expect lifetime medication therapy and the risks of worsening only increase with each successive surgery as revision rates can range from 9% to 45% [5-7].

The purpose of this case report is to add options for clinicians, physicians, and therapists to improve both subjective and objective measures using specific spine and postural Mirror Image® (MI) therapies [8-14]. This simple, inexpensive, and highly studied rehabilitation protocol provides an efficient, repeatable, and reliable method to improve sagittal and coronal spinal balance and abnormalities and reduce bodily pain HRQoL suffering and GBD [8-14]. Spine conditions such as back pain, neck pain, and headaches are amongst the largest contributors to GBD and Chiropractic BioPhysics® (CBP®) (Chiropractic BioPhysics, Eagle, USA) treatment offers an economical and effective approach to multiple spine conditions including PSPS. The aim of this study is to present clinicians, physicians, surgeons, and other therapists who treat spine pain as a possible treatment option for even complex conditions such as FBSS.

## Case presentation

On October 25, 2022, a 50-year-old female presented to a spine clinic in Gillette, Wyoming, USA. There were multiple subjective complaints including headaches, neck, middle back, lower back, and bilateral hip pains with associated stiffness. There was also a report of poor circulation in the hands with coldness, frequent headaches associated with blurred vision, frequent motion sickness, and the pains interrupted her sleep.

The patient reported injuring her back in 2004 while lifting a large microwave oven. She reported severe LBP and bilateral leg pain and received some physical therapy. Following treatment, she reported that she still had pain and dysfunction with bending and lifting, but she returned to work for financial reasons without further treatment for several years. Over the years she would have episodes of LBP with bilateral radiculopathy sometimes past the knee that was associated with muscle spasms and the feeling of her spine being ‘locked up’ which would cause severe disability for a time and would slowly resolve. After three years of suffering, she underwent a pedicle screw fixation of L5/S1 in 2007 due to degenerative disc disease at that level.

After the patient was cleared to return to work, she had 10 years of successful workability, but her occupation as a heavy machine and large equipment operator at a coal mine caused her to have exacerbations and in 2014 she underwent a revision of the previous L5/S1 fusion and with a discectomy at L3/L4 and L4/L5 coupled with expanded pedicle screw fixation from L3-S1. The patient’s PROs were slightly improved following the procedure, however, the pain persisted, and she frequently experienced episodes of severe pain and muscle spasms in the lumbar paraspinal muscles into the buttocks. Since this second surgery, she used over-the-counter (OTC) medication and sought traditional chiropractic spinal manipulative therapy (SMT) that gave her only temporary relief but did not allow a return to normal activities of daily living (ADLs).

Posture assessment showed a right lateral translation of her head in relation to her thorax, an anterior thoracic translation of the thorax over the pelvis, and a left posterior rotation of the pelvis compared to the feet. Spinal palpation of the paraspinal muscles indicated tight and tender fibers with increased pain in the mid-cervical spine, apex of the sagittal thoracic curve, and throughout the lumbar spine and bilateral hips. Orthopedic tests revealed cervical and lumbar compression tests, as well as hip joint compression tests, were positive for pain. Cervical range of motion (ROM) was restricted in all planes of motion and cervical flexion, left lateral flexion and right rotation also elicited pain centrally. Lumbar ROM found restriction in all planes tested and was reported painful in flexion and extension as well as left lateral flexion and right rotation with bilateral lumbar paraspinal pain. Sensory dermatome testing revealed hypoesthesia at L4-S1 levels.

The cervical and lumbar spinal areas were reported to average a 3/10 (range 1-10) and 5/10 (range 3-9) on an 11-point numerical pain rating scale (NPRS), respectively. The headache disability index (HDI) [15] scored 46/100, indicating moderate disability. The neck disability index (NDI) [16] scored a 26% indicating severe disability. The revised Oswestry LBP disability questionnaire (ODI) [17] scored 50%, equating to severe disability. HRQoL was assessed by the short-form 36 (SF-36) [18] and scores were below norms for 7/8 indices (Table 1).

**Table 1:** Health-related quality of life scores using the short-form 36 (SF-36) questionnaire.

Date	Health	Physical	Physical	Emotional	Social	Mental	Bodily	Energy
	Perception	Function			Function	Health	Pain	Fatigue
Normal	72	84	81	81	83	75	75	61
10/25/2022	92	70	25	0	63	48	45	30
1/27/2023	87	100	100	100	100	84	78	70
7/19/2023	87	95	100	100	88	76	80	35
Overall Change	-5	25	75	100	25	28	32	5

**Figure 1:**
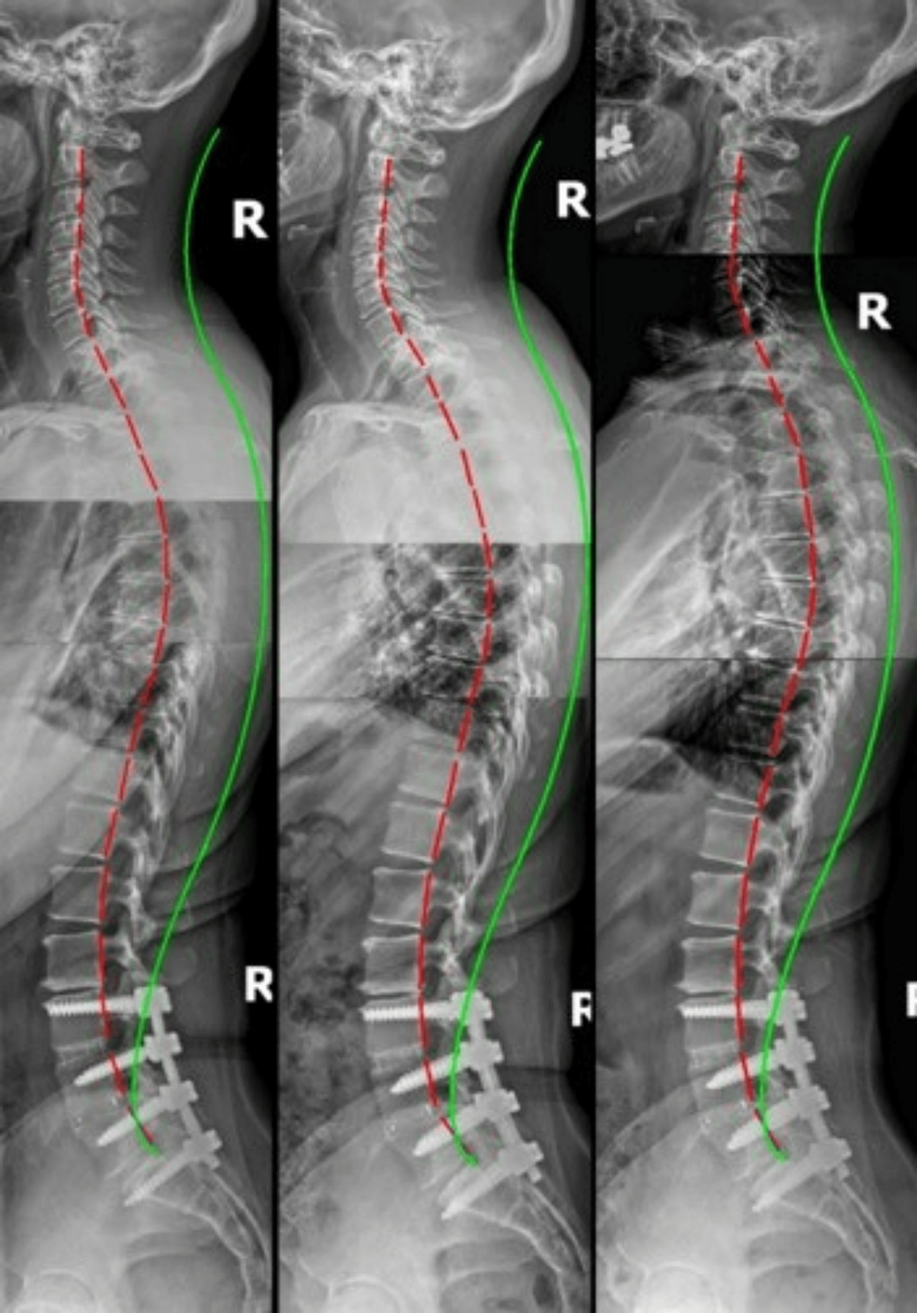
Lateral spinal radiographs Left: Initial; Middle: Post-treatment; Right: Six-month follow-up. Green lines indicate ideal alignment; red lines highlight the patient’s spinal position.

**Figure 2:**
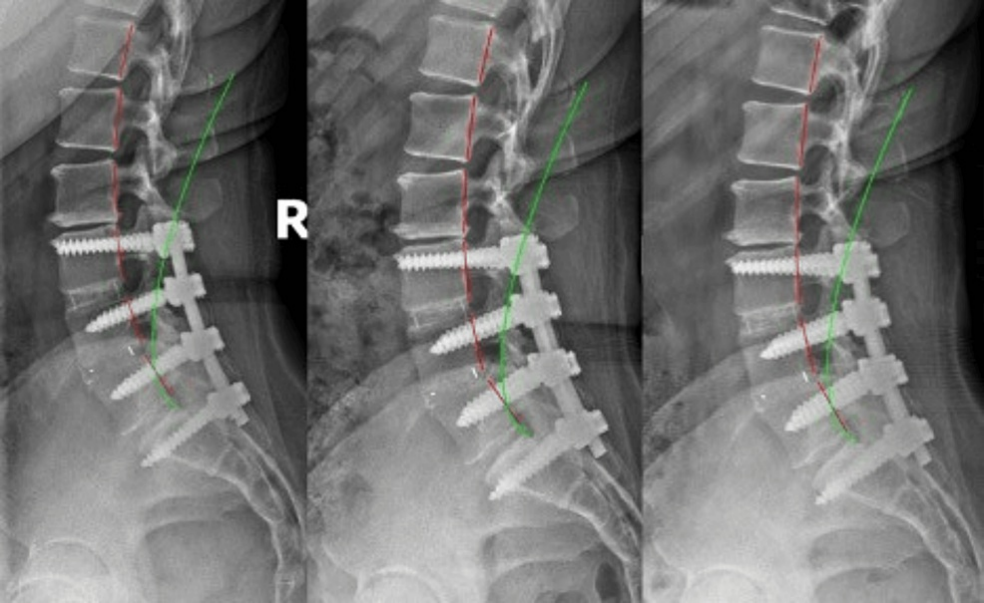
Lateral lumbar X-rays Left: Initial; Middle: First post-treatment; Right: Six-month follow-up. Notice surgical hardware from L3-S1. Green lines indicate an ideal curve; red lines highlight the patient’s spinal alignment. Note the reduction of the horizontal distance between the top of the green line and the posterior-superior corner of the T12 vertebral body after treatment.

**Figure 3:**
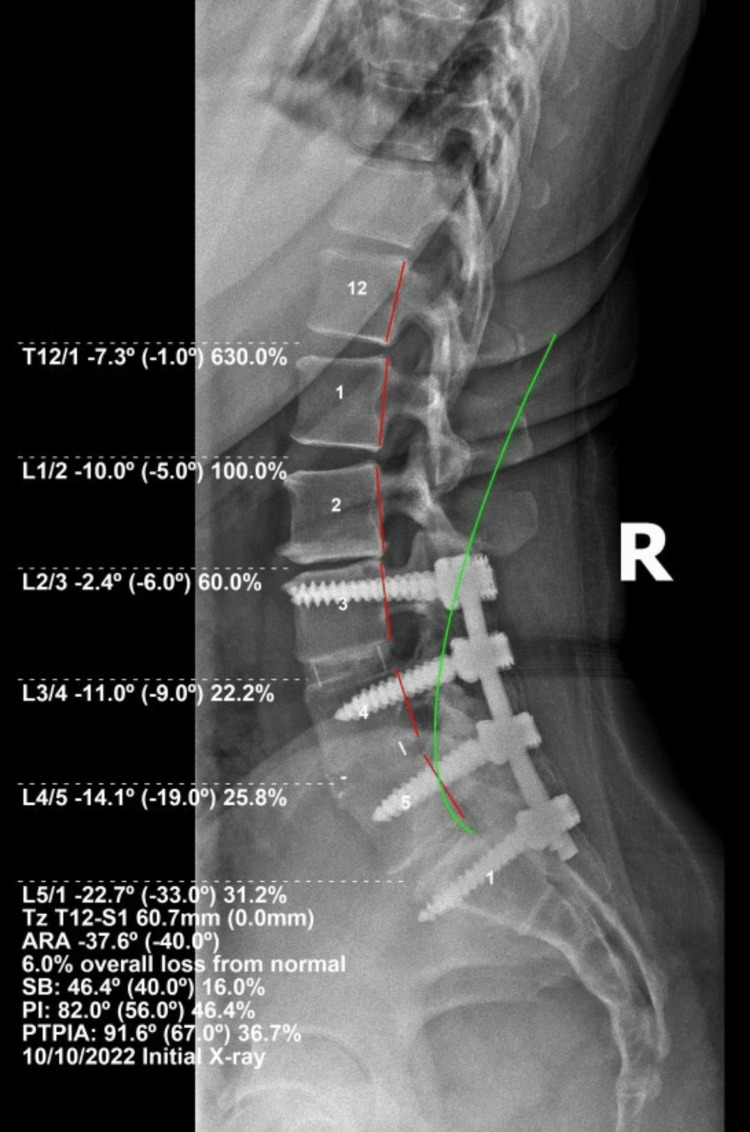
Initial lateral lumbar radiograph with PostureRay Biomechanical assessment.

Full spine radiographs (Figure 1) were obtained and analyzed using the PostureRay™ EMR (Trinity, FL, USA) which has been shown to be a valid method for capturing biomechanical parameters for biomechanical treatment [19-21]. This system also uses the Harrison posterior tangent method for sagittal alignment analysis [22-27]; this line-drawing approach has established reliability. Notably, the patient had a forward sagittal vertical axis (SVA) from T1-S1 of 60.0mm, loss of cervical lordosis (-14.2° vs. 20-42°), a reduced atlas plane line (APL: -22.3° vs. 24-29° normal), minimal anterior head translation (5mm), loss of thoracic kyphosis (35.8° vs. 44°), a 37.6° lumbar lordosis (vs. 40° ideal), a forward translation of the thorax relative to the pelvis (60.1mm vs. 0 ideal), a 46.4° sacral base angle (SBA; vs. 40° ideal) [19-27]. Also noted is the surgical instrumentation visualized from L3-S1, and this appeared to be stable with no obvious rod/screw failure and no apparent loosening (Figure 2). Figure 3 demonstrates the lateral lumbar biomechanical assessments.

The patient opted for conservative CBP® therapy [8-14] and was treated 12 times from 10/25/2022 to 1/27/2023. CBP typically involves MI® exercises, spinal manipulation to improve posture, as well as unique spinal traction methods. The patient performed exercises using a neck strengthening device (Prolordotic™ Spinal exerciser Circular Traction LLC., Huntington Beach, CA, USA) which involved neck hyperextension exercises against resistance while sitting and hunching the middle back (2 minutes), core strengthening ‘mountain-climber’ plank-style exercises (2 minutes), hip abductions with a resistance band as well as wearing Meyer’s weighted thoracic posture device (Circular Traction LLC., Huntington Beach, CA, USA) (8 pounds) to induce a posterior thoracic translation and rounding of the shoulders while walking for 4 minutes. All the exercises were performed on a Power Plate® whole body vibration (WBV) device (Power Plate, IL, USA) to increase their intensity.

**Figure 4:**
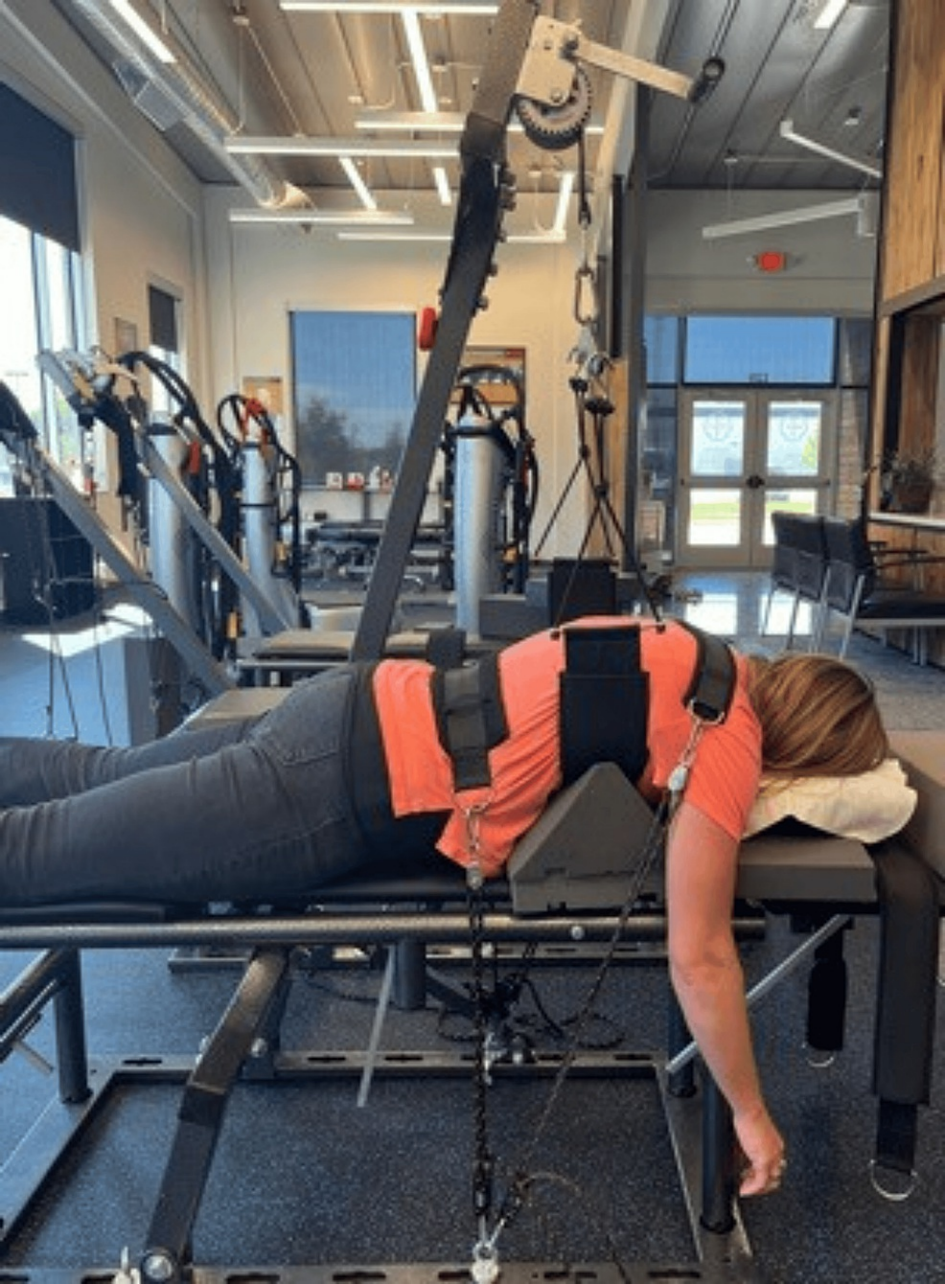
Mirror Image traction. The patient is prone to a force used to increase thoracic curvature and induce posterior thoracic translation.

There were two MI® traction procedures used to improve cervical lordosis. The patient was seated with a fulcrum pulling forward at the mid-cervical level with up to a 25-pound weight, simultaneously, the head was pulled backward with a static pull [9]. Second MI® traction was a prone position with the lumbar spine immobilized with a fabric strap and a support fulcrum was placed under the ribcage to move the thoracic spine posteriorly and the upper thoracic spine was immobilized with a posterior-to-anterior fabric strap (Figure 4). A mechanical force was used to increase the thoracic curvature as well as to induce a posterior translation of the thorax and lessen abnormal loads on the discs and other spinal and paraspinal structures. Both tractions were performed starting at 2-5 minutes and working up to 12-15 minutes per session in order to induce viscoelastic creep in the ligamentous structures [10].

The patient was treated initially for 12 sessions over a 3.5-month period. The patient has also been prescribed a home treatment regimen of cervical traction with a medium Denneroll™ traction block (Denneroll Spinal Orthotics, Wheeler Heights, NSW, Australia) as well as the cervical extension exercises previously described at a frequency of 100 repetitions daily. A follow-up assessment was performed approximately nine months after initiating care. The patient consented to the publication of this report.

Results

Following care, the LBP was reported as 80% improved, bilateral hip pain 100% improved, cold feet 50% improved, neck pain into shoulders 90% improved, headaches 80% improved, and difficulty sleeping due to the hip pain 80% improved. There was no reported improvement in the ringing in ears, blurred vision, or motion sickness. Physical examination showed no more muscle spasms, maximum cervical compression test showed pain localizing on the left and Yeoman’s showed localized pain on the right sacroiliac joint. Cervical ROM showed restriction without pain in flexion, left lateral flexion, and right rotation. Lumbar ROM showed restriction without pain for flexion, extension, and right rotation. Sensory testing showed L5 hypoesthesia on the right and S1 hypoesthesia also on the right. Spinal palpation indicated tight and tense muscle fibers at the levels of C4, T4, L4, and sacrum bilaterally.

The cervical and lumbar spinal areas were reported to average a 1/10 (range 0-3) and 1/10 (range 0-3) on the NPRS, respectively. Disability scales indicated dramatic improvement in all scores, 14% on the NDI (vs. 26%), 12% on the ODI (vs. 50%), and 18% on the HDI (vs. 46%). There were also improvements in 7/8 indices on the SF-36 (Table 1). The radiographic assessment showed a 16° improvement in cervical lordosis (-30.2° vs. -14.2°), a 6.9° increase in APL (-29.2° vs. -22.3°), a 7.8° increase in thoracic kyphosis (35.8° vs. 43.6°) and a reduction in anterior thoracic translation (55.5mm vs. 60.1mm). The SVA remained similar (61.8mm vs. 60.0mm). Following the initial treatments, the patient continued to perform the home traction and postural stabilizing exercises daily but did not continue treatments in-office.

A follow-up assessment six months following the last assessment (nine months after starting care) demonstrated the maintenance of improved outcomes. Subjective rating indicated that the burden of complaints was LBP 70% improved, bilateral hip pain 70% improved, cold feet 50% improved, neck pain into shoulders 80% improved, headaches 60% improved, difficulty sleeping due to hip pain 60% improved, with no improvement in ringing in ears, blurred vision and motion sickness. Palpation showed tight and tender muscle fibers at the levels of C5, T7, L5, and the sacrum. There were no muscle spasms. Orthopedic tests were all negative. ROM testing showed restriction without pain for cervical flexion and lumbar extension. Dermatome testing was negative.

The cervical and lumbar spinal areas were reported to average a 1/10 (range 1-5) and 2/10 (range 1-4) on the NPRS, respectively. Disability scale scores showed 6%, 14%, and 16% on the NDI, RODI, and HDI. The minimal clinically important difference (MCID) for the NDI is 14-15%. The SF-36 scores showed that the patient was above the normal values for 7/8 indices with the exception of the energy/fatigue scale regressing to 35/100. Radiographic analysis showed the maintenance of the cervical lordosis (-27.7° vs. -30.2°), APL (-30.3° vs. -29.2°), a regression of the thoracic kyphosis (43.6° vs. 37.7°), a further reduction in anterior thoracic translation (52.3mm vs. 55.5mm), and a reduction in the SVA (39.5mm vs. 61.8mm) [20-27].

## Discussion

This case demonstrates that non-surgical spinal rehabilitation procedures using MI® spine techniques can improve subjective symptom reporting, PROs, HRQoL as well as spine biomechanical parameters as measured on the sagittal radiographs in patients having previous lumbar spine surgery. These results are consistent with other conservative CBP® studies [8-14] including case reports and case series, RCTs, modeling and biomechanical studies, and others in the approximately 340 CBP® published studies. Many of these show unusual cases that are unique and offer insight into possible treatments for distinct conditions. Pedicle screw fixation has been in use for 80 years and although is it successful often, failure rates are found to be 34% to 64% [28,29]. This failure can be due to failure of single or multiple screws and/or rods, loosening of the hardware, poor placement, poor purchase of bone, or simply due to poor post-surgical improvement in sagittal and coronal balance with related adjacent segmental degeneration, osteoporosis, and other arthritic and degenerative pathologies [1,4-7,29].

It is important to note that revision and re-operation come with greater risks to the patient and are associated with greater failure rates versus a single, initial surgical procedure [5]. Patients can be classified as having failed low back surgery syndrome if PROs are not improved after the fusion should be stable (i.e. 3-12 months) [7]. If the HRQoL and the sagittal and coronal balance are not improved and/or there is hardware failure, infection, or vascular abnormalities due to the surgery, a diagnosis of FBSS/PSPS is requisite [30]. Poor low back spine surgical outcomes are treated with many conservative drug therapies including non-steroidal anti-inflammatories and over-the-counter pain relievers as well as prescription opioids and steroids, and physical therapies [2,3,5,27-29].

In the case reported here, the patient underwent two posterior approach pedicle screw fixation procedures. Despite these invasive interventions, the PROs were not improved until she received non-surgical spinal rehabilitation with CBP® methods. Very recent research and nomenclature studies recognize FBSS and adult spine deformity (ASD) as a new category of persistent spine pain syndrome (PSPS-T1/T2) [30]. The importance of multidisciplinary and a holistic approach is suggested in the treatment of PSPS-T1/12, and CBP® methods may be a good match to address this need as described in the present case.

It is important to appreciate that spine radiography with a high-quality structural analysis EMR is critical in order to properly diagnose a patient’s biomechanical condition, enable better differential diagnosis, and provide feedback on treatment progress [31-35]. Indeed, X-ray diagnosis is more accurate than non-radiographical diagnosis [36]; this is obviously more important for patients having a history of spine surgery. Further, plain film radiography for spine diagnosis and care is safe, economical, and carries little risk of additional harm, and physicians who do not use routine upright full spine X-rays will miss potential treatment options and could worsen the patient’s risk of ASD and further contribute to the GBD [37-40].

## Conclusions

We have presented a case of a single patient improving with this protocol. Results are consistent with prior studies. Sagittal balance, coronal balance, spinal alignment improvement as well as measurable improvements in HRQoLs are desirable clinical outcomes for clinicians who treat spinal pain and this novel CBP® protocol consistently demonstrates these improvements. The treatment protocol involved a multi-modal approach making definitive individual treatment modality contributions indistinguishable from the total effect of the program. Regardless, this case contributes to the growing body of evidence that the CBP® technique may be effective in improving subjective and objective outcome measures both short- and long-term. Larger studies including randomized clinical trials are necessary to make firm conclusions on the efficacy of CBP® for PSPS/FBSS and other conditions.
